# Hypoglycemic and Hypolipidemic Swords: Synthesis and *In-vivo *Biological Assessment of 5-benzylidene-2,4-thiazolidinediones

**DOI:** 10.22037/ijpr.2021.114969.15131

**Published:** 2021

**Authors:** Vijay Patil, Neha Upadhyay, Kalpana Tilekar, Hardik Joshi, C S Ramaa

**Affiliations:** *Department of Pharmaceutical Chemistry, Bharati Vidyapeeth’s College of Pharmacy, Navi Mumbai, India.*

**Keywords:** Diabetes, Hypoglycaemic, Hypolipidemic, PPARγ, thiazolidinediones

## Abstract

Thiazolidinedione (TZD), being a privileged scaffold, has been known as a significant structural moiety of antidiabetic drugs. TZD has been known to improve glycaemic control in type 2 diabetes mellitus (T2DM) by increasing insulin sensitivity in the body. A novel series of 5-benzylidene 2,4-thiazolidinedione derivatives were designed, synthesized (V1-V28), and structurally confirmed by different spectroscopic techniques such as FTIR, ^1^H NMR, ^13^C NMR, and Mass spectrometry. Upon the safety assessment of the synthesized molecules in non-transformed hepatocytes by MTT reduction assay, these were found non-toxic. These derivatives were then further evaluated for their antihyperglycemic and antihyperlipidemic properties in a high-fat diet and low dose of streptozotocin-induced diabetic rats. Altogether, seven biochemical parameters were analyzed, namely blood glucose, triglycerides, cholesterol, creatinine, blood urea nitrogen, HDL-cholesterol, and glycosylated hemoglobin in serum by standard methods. Four synthetic molecules (V2, V4, V5, and V20) possessed significant hypoglycaemic and hypolipidemic activity as compared to the positive control pioglitazone. Moreover, the histopathological studies of the heart and liver revealed no significant toxicity. Two representative compounds V2 and V4, were evaluated for their PPAR**γ** activation potential, demonstrating that they were partial PPAR**γ** agonists, thus confirming our designing hypothesis. Based on the results obtained, we assume that these compounds have the potential to be developed as future antidiabetic agents.

## Introduction

Diabetes is a chronic metabolic disease condition characterized by heightened levels of glucose in the blood as a result of either decreased insulin secretion or the development of insulin resistance. It has become a major health issue worldwide, affecting almost 285 million individuals leading to severe damage to different body organs such as the heart, eyes, kidneys, blood vessels, and nerves (1, 2). Type 2 diabetes mellitus (T2DM) is the most common, usually in adults, which leads to long-term risks such as cataracts, neuropathy, nephropathy, and atherosclerosis, which often increases the risk of myocardial infarction (3). The treatment options of diabetes have been divided into insulin therapies (insulin and its analogs) and non-insulin therapies such as insulin sensitizers (Biguanides, Thiazolidinediones), secretagogues (Sulfonylureas, Glinides), alpha-glucosidase inhibitors, incretins (such as DPP-4 inhibitors and GLP-1 receptor agonists), Pramlintide, and sodium-glucose cotransporter 2 (SGLT-2) inhibitors (4, 5). 

PPARs (Peroxisome Proliferator-Activated Receptors) belong to the nuclear receptor superfamily, which upon ligand binding, induce gene expression involved in glucose homeostasis and lipid metabolism; thus, it has been considered as a significant hallmark to treat metabolic disorders (6). Thiazolidinediones (TZDs) with antihyperglycemic activity were discovered after the discovery of ciglitazone and further developed glitazones (7). However, some of these drugs were abandoned during the clinical trials as they had been associated with toxicity due to the full PPARγ agonistic activity (8–11). Therefore, new drugs with partial, weak, or moderate PPARγ agonistic activity are being designed and developed to retain the beneficial effects while reducing the detrimental ones (12–16). The 5-benzylidene-thiazolidin-2,4-dione derivatives have been widely explored for reducing glucose and PPARγ modulation (17, 18). It is evident from the above observations that steps are being taken in the direction for the production of independent or partial agonists of PPARγ. Thus, in the present work, we have synthesized a library of novel 5-benzylidene 2,4-thiazolidinedione derivatives (V1-V28) in search of less toxic derivatives of TZD and performed structural characterization of these compounds. All these TZD derivatives were then evaluated to determine theirs *in vivo* antidiabetic activity.

*Rationale of designing*

**Figure 1:**
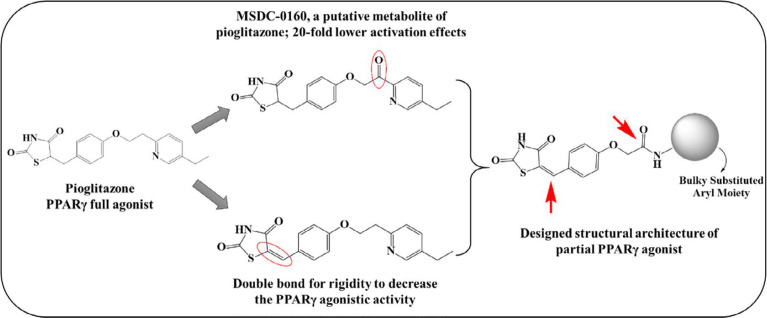
Designing considerations of partial PPARγ agonists

It has been assumed that the adverse effects associated with glitazones are due to their full agonistic potential with PPARγ (10, 19). The literature evidence indicates that the adverse effects could be minimized by using partial agonists of PPARγ receptor, which may be due to lowered transcriptional capabilities, bypassing the ill effects associated with the full agonistic activity. This gave us an impetus to develop novel chemical entities with possible partial agonistic activity to dodge these toxicity issues. Herein, we considered pioglitazone as the prototype and made changes to the non-pharmacophoric part with the intention of conferring partial binding capability to the molecules. We retained the pharmacophore moiety, *i.e*., TZD, with different structural modifications, specifically at the lipophilic tail. The first modification was introducing rigidity into the structure by adding benzylidene double bond or unsaturation at the TZD site, which may restrict the rotation of the moiety, making it less accessible to the receptor. The second modification was introducing an amide soft spot similar to that of MSDC-0160 (the putative metabolite of pioglitazone), which was proposed to have 20-fold fewer activation effects at the receptor site as compared to pioglitazone (20) (Figure 1). The introduction of the amide modification would divert the metabolism away from the TZD ring, again serving to reduce toxicity. With these designing considerations, we have previously reported a series of chemical compounds with different structural modifications at the lipophilic portion with promising primary antidiabetic (21) and anticancer (22, 23) potential; however, the PPARγ activation capacity was undetermined. With increasing interest in partial PPARγ agonists to overcome the detrimental effects associated with full agonists, it was worthwhile taking this series further with more number of potential derivatives incorporating structural variations. In this structural skeleton of the proposed partial PPARγ agonists, we have incorporated differently substituted aromatic/heteroaromatic moieties at the lipophilic tail portion to obtain various derivatives (V1-V28).

## Experimental

*Chemistry*

Each step of the reactions was observed for completion with the use of pre-coated silica plates (Merck Silica Gel 60 F254) thin layer chromatography (TLC). The melting points of all the intermediates and final derivatives were recorded on an automated melting point apparatus (VEEGO) and are uncorrected. IR spectrum was recorded with the help of Shimadzu FTIR- 8400S by direct sampling technique in the scanning range of 500-3600 cm^-1^. The ^1^H and ^13^C NMRs were obtained by Bruker 400 MHz and 100 MHz instruments, respectively, using DMSO-d6 as a solvent. The chemical shift values are expressed in ppm (δ). 

*Cell viability assessment: Evidence on toxicity in untransformed hepatic cells*

The effect on the viability of untransformed hepatocytes was determined as described previously (24). Briefly, the whole rat liver was homogenized in 0.32 M sucrose medium and centrifuged at 1073 ×g for 10 min. The supernatant was separated and recentrifuged for 15 min at 17,172 ×g. The pellets were further resuspended in 40 mL of HEPES buffer. The cell viability of hepatocytes was assessed by MTT [(3-(4,5-dimethylthiazol-2-yl)-2, 5-diphenyltetrazolium bromide)] assay. The hepatocytes (400 µL) were incubated for 2 h at 37 ◦C along with 100 µM compound concentration. Further, MTT (8 µL, 5 mg/mL) was added, and plates were incubated for 1 h at 37 ◦C. The formed formazan was estimated by UV spectroscopy at 570 nm. Results were presented as % of MTT reduced in comparison to control values. The assay was performed in triplicates, and the result values are expressed as the mean ± SEM.

*Antidiabetic assessment*

*Animals*

 The animal was procured with the procedure described elsewhere (21). The SD rats (Male Sprague–Dawley, 160–180 g) were obtained from Bharat serum, Road no. 27, Plot no. 371-372, Wagle Industrial Estate, Thane, 400604. The animals were stored in polypropylene cages (three rats per cage) and preserved at room temperature (22 ± 2 ◦C) and under humidity (55 ± 5 %) in a 12:12 h cycle (light and dark). All SD rats were fed with NDP (normal pellet diet) before dietary manipulation. Prior permission was obtained from the animal ethics committee CPCSEA, Government of India (registration number 762/-3/C/CPCSEA) for conducting the animal experiments.

*Low dose STZ with HFD-fed rat model*

 This model is suitable for the pharmacological evaluation of test compounds as it replicates the metabolic characteristics of T2DM. The SD rats were administered a high-energy diet of 20% sucrose and 10% lard along with a single injection of STZ (30 mg/kg body weight). After 4 weeks, changes in body weight are recorded, and levels of Glucose, Triglycerides, Cholesterol, Creatinine, Blood Urea Nitrogen, HDL-Cholesterol, Glycohemoglobin in serum were analyzed by standard methods.

*Procedure*

 Two weeks after the dietary manipulation, rats were induced with hyperglycemia by use of a single i.p. injection of STZ (30 mg/kg) in 0.1 M and 4.5 pH citrate buffer; whereas, in control rats, 0.1 M and 4.5 pH vehicle citrate buffer was injected (1 ml/kg, i.p.). During this period, animals were also measured for their feed and water intake. The SD rats were continued on their respective diets till the study ended. The body weight and biochemical estimations (blood Glucose, triglycerides, cholesterol, creatinine, blood urea nitrogen, HDL-cholesterol, and glycosylated hemoglobin) were determined in the serum, and 7, 14, and 21 days after the dose, blood samples from the animals were withdrawn in light ether anesthesia from the retro-orbital venous plexus. The serum was centrifuged to determine the level of glucose. The SD rats with more than 300 mg dl^-1 ^glucose levels were selected for further experiments. Hyperglycaemic rats were divided into twenty-three groups (6 rats in each group). Apart from twenty groups for twenty synthesized compounds, one group served as a vehicle or normal control (received only citrate buffer), one group was diabetic control, and another group served as standard diabetic control, which received pioglitazone as a reference drug. Drug treatment was initiated 48 h after STZ injection (at which hyperglycemia was confirmed). After 24 h of the last dose of drug treatment, the serum levels of Blood Glucose, triglycerides, cholesterol, creatinine, blood urea nitrogen, HDL-cholesterol, and glycosylated hemoglobin were established using specific diagnostic kits.

*Blood collection*

 The blood sample was withdrawn from the retro-orbital plexus of SD rats by using capillary tubes and transferred into heparin (20 l, 200 IU mL^−1^) containing Eppendorf tubes. The plasma was collected by centrifugation for 5 min at 5000 rpm and was analyzed for glucose (GOD-POD), total cholesterol (CHOD-POD) and triglycerides (GPO-POD), and other biochemical parameters levels using colorimetric diagnostic kits (Accurex Biomedical, Thane, India).

*Statistical analysis*

The results have been expressed in mean ± SEM. The Student’s *t*-test (unpaired) was used for the analysis of the data between the two groups; whereas, a one-way ANOVA test followed by Tukey’s test was employed for more than two groups. The value of *p* < 0.05 was assumed statistically significant (25).

*Histopathology*

After 21 days of treatment, three novel compounds with maximum hypoglycaemic and hypolipidemic activity compared to other derivatives were selected for histopathological study to determine the toxicity effect. The animals were euthanized by CO_2_ exposure, and histopathology was performed for the heart and liver. The liver and heart were preserved in 20% formalin immediately after removal from the animal.

*PPAR-γ transactivation assay*

To determine the PPARγ transactivation capabilities of compounds V2 and V4, an ELISA test was carried out against pioglitazone as positive control by using a similar procedure as mentioned by Joshi *et al. *(24). Nuclear extract was collected after treatment with the test compound and positive control, the Abcam’s ELISA kit was used to determine the PPARγ transcriptional activity.

## Results and Discussion

*Chemistry*

Scheme I illustrate the synthesis of intermediates and final compounds. Synthesis of final compounds V1-V28 proceeded via three steps (22, 26, and 27). Step I was Knoevenagel condensation of 2,4-TZD and 4-hydroxy benzaldehyde to yield 5-(4-hydroxybenzylidene)-2,4-thiazolidinedione. The Knoevenagel compound (5-(4-hydroxybenzylidene)-2,4- thiazolidinedione) was common for the synthesis of all the final compounds. This intermediate was further reacted with different chloroacetylated amines (M1-M28) to obtain the final step compounds (V1-V28). The chloroacetylated intermediates (M1-M28) were prepared by the procedure mentioned elsewhere (28, 29). Structures of all the final compounds were confirmed by different spectroscopic techniques such as FTIR, NMR (^1^H & ^13^C), and Mass spectrometry (characterization of other compounds of this series has been previously reported by us (22, 23)).

The structure of the chloroacetylated aryl/heteroaryl amine intermediates were confirmed by FTIR and ^1^H-NMR. The FTIR of the intermediates chloroacetylated aryl/heteroaryl amines demonstrated characteristic band in a region of 1700-1660 cm^-1 ^for (C=O)-NH and 3350 cm^-1^ for -NH- group. The chloroacetylated moieties were confirmed by the singlet of -CH- protons at 4.0-4.8 δ ppm in ^1^H-NMR spectra; whereas, –CH_2_-O- linkage for the final molecules were evident by the -CH_2_ protons found in the region of 4.6 δ ppm as a singlet. Amide protons showed singlet at around 9 δ ppm. The ^13^C-NMR spectrum displayed characteristic peaks in the region of 65-68 ppm for –CH_2_O-, and carbonyl peaks observed between 155-168 ppm. The molecular mass of the final compounds was confirmed by mass spectrometry, which revealed the respective molecular ion (M+)/M+H/M-H peaks of the synthesized compounds.

*N-(5-Chloro-pyridin-2-yl)-2-[4-(2,4-dioxo-thiazolidin-5-ylidenemethyl)-phenoxy]-acetamide (V1)*

 Pale yellow. Yield – 58%. M.P. 256-258 °C. FTIR - 3381 N-H stretching; 1790, 1649 C=O stretching; 1595 C=N; 1278, 1060 Ar-C-O. ^1^H NMR (300 MHz, δ, ppm, DMSO-d_6_): 11.1 (bs, 1H, -CO-NH-CO-), 8.4 (s, 1H, -NH-), 7.8-7.9 (m, 4H, benzylidene proton, aromatic protons), 7.5 (d, *J = *8.4 Hz, 2H, aromatic protons), 6.8 (d, *J = *8.7 Hz, 2H, aromatic protons), 4.5 (s 2H, methylene protons). ^13^C NMR (75 MHz, DMSO-d_6_, δ): 167.7(C=O), 167.1(C=O), 165.3(C=O), 160.4, 150, 146.2, 137.8, 134, 132.4, 125.5, 123.5, 116.3, 116, 114.5, 66.5. Theoretical mass: 389.02, LC-MS (m/z, I %): 390 [(M+H)^+^, 60%].

*2-[4-(2,4-Dioxo-thiazolidin-5-ylidenemethyl)-phenoxy]-N-(3-methoxy-phenyl)-acetamide (V2)*

 Pale yellow. Yield – 79%. M.P. 220-222 °C. FTIR 1681, 1735, CO-NH-CO stretching; 1147 C-O stretching; 3199 N-H stretching. ^1^H NMR (300 MHz, δ, ppm, DMSO-d_6_): 10.4 (s 1H, -CO-NH-CO-), 7.8 (s, 1H benzylidene proton), 7.51-7.54 (d, *J = *9 Hz 2H, aromatic protons), 7.05-7.32 (m, 3H, aromatic protons), 6.92-6.95 (d, *J = *8.7 Hz 2H, aromatic protons), 6.64-6.67 (m, 2H, aromatic protons), 4.49 (s, 2H, methylene protons), 3.7 (s, 3H, methyl protons). ^13^C NMR (75 MHz, DMSO-d6, d):167.1 (C=O), 165.4 (C=O), 163.7(C=O), 160.2, 159.5, 139.5, 134, 132.5, 129.4, 123.6, 116.3, 116.2, 111.2, 109.1, 104.7, 67.4, 54.8. Theoretical mass: 384.08, LC-MS (m/z, I %): 385 [(M+H)^+^, 100%].

*2-[4-(2,4-Dioxo-thiazolidin-5-ylidenemethyl)-phenoxy]-N-p-tolyl-acetamide (V3)*

 Pale yellow. Yield – 70%. M.P. 264-266 °C. FTIR - 1635, 1790 CO-NH-CO stretching; 1153,1045 C-O stretching; 2937, 3199, 3321 N-H stretching and C-H stretching. ^1^H NMR (300 MHz, δ, ppm, DMSO-d_6_): 10.3 (s, 1H, -CO-NH-CO-), 7.89 (s, 1H benzylidene proton), 7.52-7.54 (d, *J = *6.6 Hz 2H, aromatic protons), 7.42-7.44 (d, *J = *6.3 Hz 2H, aromatic protons), 7.11-7.13 (d *J = *6.3 Hz 2H, aromatic protons), 6.93-6.95 (d, *J = *6.3 Hz 2H, aromatic protons), 4.48 (s 2H, methylene protons), 2.25 (s 3H, methyl protons). ^13^C NMR (75 MHz, DMSO-d6, d):167.1 (C=O), 165.3 (C=O), 164.7(C=O), 160.9, 144.9, 142.5, 134.2, 132.5, 124.7, 123.3, 118.8, 116.4, 115.5, 60.9, 20.9. Theoretical mass: 368.08, LC-MS (m/z, I %): 370 [(M+2H)^+^, 88%].

*N-(4-Bromo-2-fluoro-phenyl)2-[4-(2,4-dioxo-thiazolidin-5-ylidenemethyl)-phenoxy]-acetamide (V4)*

 Pale yellow. Yield – 74%. M.P. 258-260 °C. FTIR - 1639 C=O stretching; 3043, 3188 N-H stretching, aromatic C-H stretching; 1299 C-N stretching; 570 C-Br stretching. ^1^H NMR (300 MHz, δ, ppm, DMSO-d_6_): 10.3 (s 1H, -CO-NH-CO-), 7.83-7.89 (m 2H, benzylidene proton, aromatic proton), 7.5-7.65 (m, 3H, aromatic protons), 7.36-7.39 (d, *J = *8.7 Hz, 1H, aromatic proton), 6.92-6.95 (d, *J = *9 Hz, 2H, aromatic protons), 4.57 (s 2H, methylene protons). ^13^C NMR (75 MHz, DMSO-d6, d):167.1 (C=O), 165.3 (C=O), 164.5(C=O), 160.2, 134, 132.5, 127.3, 123.6, 116.3, 116.1. Theoretical mass: 449.97, LC-MS (m/z, I %): 448.1 [(M-2H)^+^, 65%].

*N-(6-bromo-2,4-difluorophenyl)-2-[4-(2,4-dioxo-thiazolidin-5-ylidenemethyl)-phenoxy]-acetamide (V5)*

 Pale yellow. Yield – 70%. M.P. 266-268 °C. FTIR - 1679,1735 CO-NH-CO stretching; 1149,1095 C-O stretching; 3222, 3261 N-H stretching; 1240 C-N stretching. ^1^H NMR (300 MHz, δ, ppm, DMSO-d_6_): 10.3 (s 1H, -CO-NH-CO-), 8.16 (s 1H, aromatic proton), 7.89 (s 1H benzylidene proton), 7.77-7.79 (d, *J = *8.1 Hz 1H, aromatic proton), 7.51-7.58 (m, 3H, aromatic protons), 6.92-6.95 (d, *J = *9 Hz 2H, aromatic protons), 4.6 (s 2H, methylene protons). ^13^C NMR (75 MHz, DMSO-d6, d):167 (C=O), 165.3 (C=O), 165.2 (C=O), 160.2, 133.9, 132.8, 132.5, 123.6, 116.3, 116.2, 104.2, 51.5. Theoretical mass: 467.96, LC-MS (m/z, I %): 467 [(M-H)^+^, 89%].

*2-(4-((2,4-dioxothiazolidin-5-ylidene)methyl)phenoxy)-N-(4-methoxyphenyl) acetamide (V6)*

 Pale yellow. Yield – 74%. M.P. 272-274 °C. FTIR-1674, 1731 CO-NH-CO stretching; 1146, 1176 C-O stretching; 3317 N-H stretching. ^1^H NMR (300 MHz, δ, ppm, DMSO-d_6_): 10.2 (s 1H, -CO-NH-CO-), 7.88 (s 1H benzylidene proton), 7.52-7.54 (d, *J = *6.0 Hz 2H, aromatic proton), 7.45-7.47 (m, 2H, aromatic protons), 6.93-6.95 (m, 2H aromatic protons), 6.88-6.91 (m, 2H aromatic protons), 4.46 (s, 2H, methylene protons), 3.72 (s, 3H methoxy proton). ^13^C NMR (75 MHz, DMSO-d6, d):167.8 (C=O), 165.9 (C=O), 163.8 (C=O), 160.7, 155.9, 133.4, 133.1, 131.9, 124.2, 121.1, 116.9, 116.8, 114.4, 55.6. Theoretical mass: 384.08, LC-MS (m/z, I %): 385.1 [(M+H)^+^, 56%].

*N-(3-Methylphenyl)-2-[4-(2,4-dioxo-thiazolidin-5-ylidenemethyl)-phenoxy]-acetamide (V7)*

 Pale yellow. Yield – 79%. M.P. 240-242 °C. FTIR - 1744,1639 C=O stretching; 1147,1248 C-N stretching; 3269 N-H stretching ^1^H NMR (300 MHz, δ, ppm, DMSO-d_6_): 10.5 (s 1H, -CO-NH-CO), 7.88 (s 1H benzylidene proton), 7.73 (s, 1H, aromatic proton), 7.51-7.54 (d, *J = *8.7 Hz 2H, aromatic protons), 7.27-7.35 (m 2H, aromatic protons), 6.92-6.95 (d, *J = *8.7 Hz 2H, aromatic protons), 4.49 (s 2H, methylene protons), 2.26 (s 3H, methyl protons). ^13^C NMR (75 MHz, DMSO-d6, d):167.1 (C=O), 165.3 (C=O), 163.8 (C=O), 160.2, 137.4, 134, 133.1, 132.5, 131.06, 130.2, 123.6, 119.8, 117.6, 116.3, 116.2, 63.3, 18.9. Theoretical mass: 368.08, LC-MS (m/z, I %): 368.8 [(M+H)^+^, 97%].

*2-[4-(2,4-dioxo-thiazolidin-5-ylidenemethyl)-phenoxy]-N-(3-fluoro-phenyl)-acetamide (V8)*

 Pale yellow. Yield – 75%. M.P. 223-225 °C. FTIR**-**1678, 1724 CO-NH-CO; 1155 C-O stretching; 3406, 3340 N-H stretching.^ 1^H NMR (300 MHz, δ, ppm, DMSO-d_6_): 10.4 (bs 2H, -CO-NH-CO-, -NH-), 7.8 (s 1H, benzylidene proton), 7.51-7.59 (m, 4H, aromatic protons), 7.14-7.19 (m 2H, aromatic protons), 6.92-6.95 (d *J = *9 Hz 2H, aromatic protons), 4.4 (s 2H, methylene protons). ^13^C NMR (75 MHz, DMSO-d6, d):167.1 (C=O), 165.3 (C=O), 165.5 (C=O), 160.2, 134.6, 133.9, 132.4, 123.6, 120.8, 120.7, 116.3, 116.2. Theoretical mass: 372.06, LC-MS (m/z, I %): 372.1 [(M)^+^, 92%].

*N-(3-Chlorophenyl)-2-[4-(2,4-dioxo-thiazolidin-5-ylidenemethyl)-phenoxy]-acetamide (V9)*

 Pale yellow. Yield – 72%. M.P. 276-278 °C. FTIR - 1695, 1743 CO-NH-CO stretching; 1226, 1089, 1172 C-O stretching; 3417, 3193 N-H stretching ^1^H NMR (300 MHz, δ, ppm, DMSO-d_6_): 10.54 (s 2H, -CO-NH-CO-, *-NH-*), 7.89 (s 1H benzylidene proton), 7.57-7.60 (d, *J = *11.4 Hz 2H, aromatic protons), 7.51-7.54 (d, *J = *8.4 Hz 2H, aromatic protons), 7.36-7.40 (d *J = *11.1 Hz 2H, aromatic protons), 6.93-6.96 (d *J = *8.4 Hz 2H, aromatic protons), 4.5 (s 2H, methylene protons). ^13^C NMR (75 MHz, DMSO-d6, d):167.7 (C=O), 165.9 (C=O), 164.5 (C=O), 160.7, 140.2, 134.5, 133.2, 131.2 124.2, 123.9, 119.1, 118.0, 116.7, 44.4. Theoretical mass: 388.03, LC-MS (m/z, I %): 389 [(M+H)^+^, 98%].

*N-(3-chloro-4-fluoro-phenyl)-2-[4-(2,4-dioxo-thiazolidin-5-ylidenemethyl)-phenoxy]-acetamide (V10)*

 Pale yellow. Yield – 73%. M.P. 283-285 °C. FTIR - 1660 C=O stretching; 3181 NH stretching; 1163 C-N stretching. ^1^H NMR (300 MHz, δ, ppm, DMSO-d_6_): 10.3 (s 1H, -CO-NH-CO-), 7.89 (s, 1H, benzylidene proton), 7.50-7.56 (m 4H, aromatic protons), 7.33-7.38 (m 1H, aromatic proton), 6.91-6.94 (d *J = *8.4 Hz 2H, aromatic protons), 4.5 (s 2H, methylene protons). ^13^C NMR (75 MHz, DMSO-d6, d): 167.0 (C=O), 165.2 (C=O), 164.1(C=O), 160.2, 133.8, 133.5, 123.7, 116.2, 111.0, 43.5. Theoretical mass: 406.02, LC-MS (m/z, I %): 406 [(M)^+^, 95%].

*N-(3,4-dimethylphenyl)-2-(4-((2,4-dioxothiazolidin-5-ylidene)methyl) phenoxy)acetamide (V11)*

 Pale yellow. Yield – 70%. M.P. 210-212°C. FTIR - 1674 C=O stretching; 1674, 1731 CO-NH-CO stretching; 1172,1089 C-O stretching; 3419 NH stretching. ^1^H NMR (300 MHz, δ, ppm, DMSO-d_6_): 10.18 (s 1H, -CO-NH-CO-), 8.26 (s, 1H NH proton), 7.87 (s, 1H, benzylidene proton), 7.48-7.51 (m 2H, aromatic protons), 7.34 (d, *J = *1.32 Hz, 1H aromatic proton), 7.24-7.27 (m, 1H aromatic proton), 7.03-7.05 (d, *J = *6.0 Hz, 1H, aromatic proton), 6.91-6.95 (m, 2H, aromatic protons), 4.47 (s, 2H, methylene protons), 2.17 (s, 6H methyl protons). ^13^C NMR (75 MHz, DMSO-d6, d): 167.1 (C=O), 165.4 (C=O), 160.2(C=O), 134.9, 134.0, 133.6, 132.6, 128.6, 123.6, 116.3, 99.49, 43.6. Theoretical mass: 382.10, LC-MS (m/z, I %): 382 [(M)^+^, 65%].

*Cell viability assessment*

**Figure 2:**
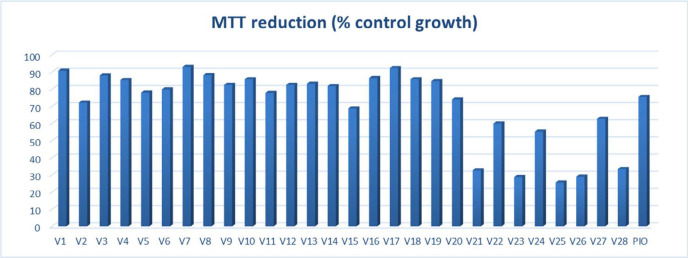
MTT cell viability assay of V1-V28 against pioglitazone

Cytotoxicity evaluation of untransformed hepatocytes was carried out by MTT (dimethylthiazol-diphenyltetrazolium bromide) colorimetric assay against test compounds and positive control pioglitazone. Recently reported TZD derivatives with anticancer potential were not evaluated for their safety assessment on normal hepatocytes (tested for their cell viability potential on cancer cells) (22); thus, altogether, these compounds (V1-V28) with pioglitazone were assessed for their capability to inhibit cell growth of normal hepatocytes (Figure 2). Out of all the test compounds, twenty of them showed more than 65% cell growth; thus, these were selected for further *in vivo *antidiabetic evaluation.

*Antidiabetic evaluation*

Antidiabetic activity of synthesized compounds was evaluated on male Sprague–Dawley (SD) rats. The low dose of STZ with HFD-fed induced hyperglycemia rat model was used to evaluate the antidiabetic potential of novel compounds (V1-V20), and pioglitazone was used as a positive control. Hyperglycemia was confirmed at 0 h in rat models by determining the level of blood glucose, cholesterol, and triglyceride using a suitable diagnostic kit. All twenty compounds revealed remarkable antidiabetic properties with a degree of variation. Percentage reduction in blood glucose, cholesterol, triglyceride, creatinine, blood urea nitrogen and Glycated Hb 7 to 21 days post oral treatment of the test derivatives was recorded, and all compounds showed a significant reduction of these parameters as compared to control diabetic rats at 15 mg/kg body weight.

**Figure 3:**
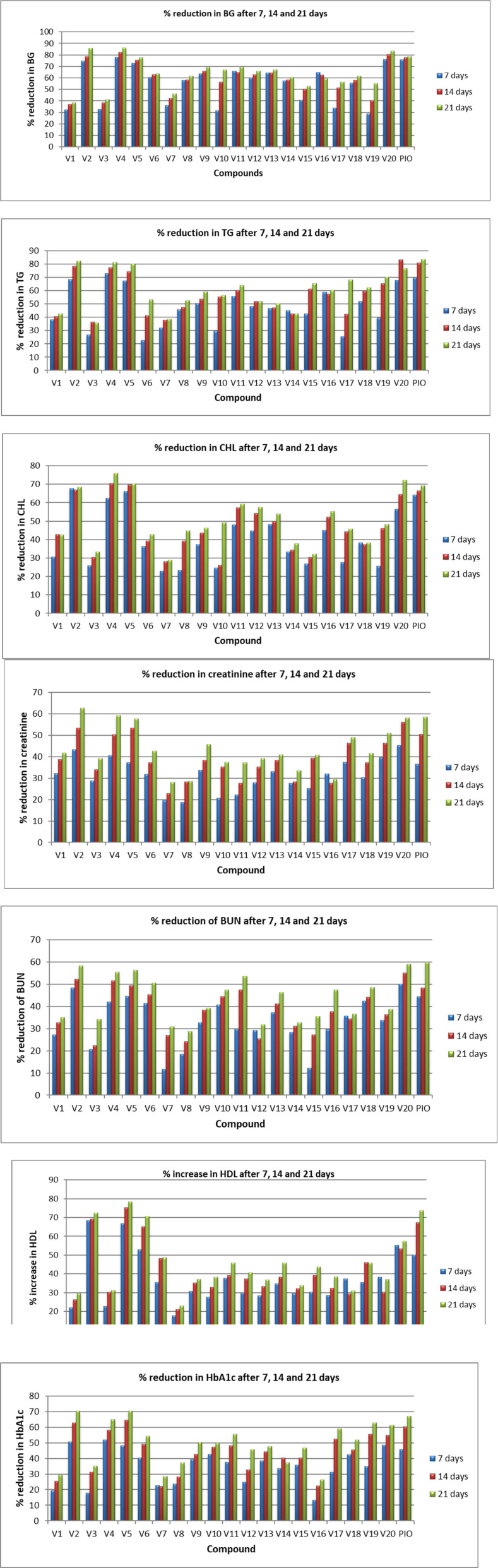
Hypoglycaemic and hypolipidemic evaluation of test compounds for parameters blood glucose, triglycerides, cholesterol, creatinine, blood urea nitrogen, high-density lipoprotein, and glycosylated hemoglobin

V2 and V5 decreased BG levels by more than 70%. These two compounds revealed comparable hypoglycaemic and hypolipidemic activity with no effect on the normal function of the kidneys. V2 and V5 showed more than 40% reduction in BG, TG, CHL, CR, BUN, and Gly. Hb in SD rats after 7 days of oral treatment. V4 demonstrated 78.30% reduction of blood glucose, 72.88% triglyceride, 62.44% cholesterol, 40.56% creatinine, 42.11% blood urea nitrogen, and 51.90% reduction in Gly. Hb after 7 days of oral treatment. In addition, V4 increased HDL level by 66.88%; however, standard PIO increased HDL level by 50.12 %. In particular, V20 exhibited 76.18% reduction of blood glucose, 67.65% triglyceride, 56.43% cholesterol, 45.31% creatinine, 50.12% blood urea nitrogen, 48.60% reduction in Gly. Hb and increase in HDL by 55.16% (Figure 3). Among all the test compounds, V4 and V20 exhibited better hypoglycaemic and hypolipidemic activity than pioglitazone in the SD rat model. After 14 days, all four derivatives (V2, V4, V5, and V20) lowered the CHL level by 60-70% in rat models. V4 and V20 decreased BG levels by more than 80%. The reduction of BG for V4 and V20 was 82.17% and 80.25%, respectively, which was more than pioglitazone (77.55%). V20 reduced TG by 83.13%, V2 and V4 decreased TG by more than 75%. In comparison, V5 showed a more than 70% reduction in both BG and TG. Favorably these four compounds showed the encouraging result in the level of HDL, BUN, CR, and Gly. Hb. The result after 21 days was more encouraging for all the test compounds and optimistic for V2 and V4. Observed blood glucose reduction for V2, V4, V5, and V20 was 85.80%, 86.27%, 83.54%, and 77.80%, respectively, while for PIO, it was 78.58%. The reduction in TG levels was 82.20%, 81.29%, 76.75%, and 80.27% for V2, V4, V5, and V20, respectively. These four compounds exhibited more than 70% reduction in the levels of CHL, which was comparable to PIO. BUN and CR level was reduced between 50 to 60% by these four derivatives. HDL level was also improved after 21 days of oral treatment of these chemical compounds. 

Altogether, the synthesized derivatives have profound effects of decreasing the BUN and Creatinine level on oral treatment, which indicates that these compounds are potential candidates for renal insufficiency. However, the remaining derivatives have shown a 30 to 50% reduction of BG, TG, CHL, BUN, CR, and Gly. Hb on 7, 14, and 21 days of oral treatment in SD rat model. 

The most common structural features observed in the synthesized derivatives endowed with potential antidiabetic activity have been described here. On the basis of the results, it was noticed that heterocyclic ring is not an essential functional moiety for a molecule to have hypoglycaemic and hypolipidemic activity. Halogenated compounds such as V4, V5, and V20 have exhibited remarkable antidiabetic potential in SD rats. In compounds V4 and V5, fluorine is present at the *ortho* position, whereas bromine is at the *para* position in V4 and at the *ortho* position in V5. Compounds containing electron-donating groups such as methyl and methoxy have demonstrated potential antidiabetic activity for, *e.g*., V2 and V20. 

*Histopathology*

**Figure 4:**
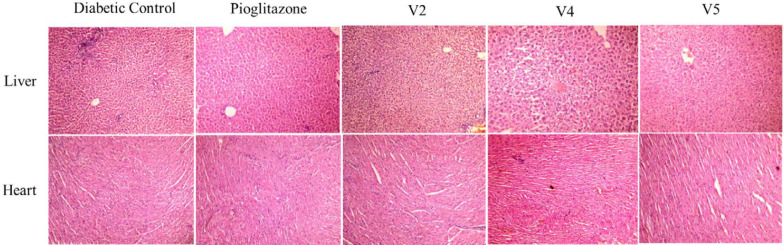
Histopathology of liver and heart on diabetic control, positive control pioglitazone, test compounds V2, V4, and V5

Analysis by light microscopy of multiple tissues from each organ was performed, and images representative of the histological profile were observed for compounds V2, V4, and V5 (Figure 4). 

Liver - The results of hepatic cells of diabetic control showed normal lobular features, which were eminent hepatocytes, normal portal triad, and the central vein was found to be prominent and congested. Pioglitazone-treated rats displayed no significant change in portal tract and vein. In test compounds, all the hepatic architectures were well preserved and appeared normal.

Heart** - **The cardiac tissues of the diabetic control displayed mild infarct lymphocyte infiltration, whereas the pioglitazone treated group reversed this morphological pattern with a more uniform and smooth arrangement of cardiac myocytes as compared to control. However, a prominent lymphocyte infiltration has been observed in all three test sample groups. 

*PPARγ transactivation assay*

**Figure 5:**
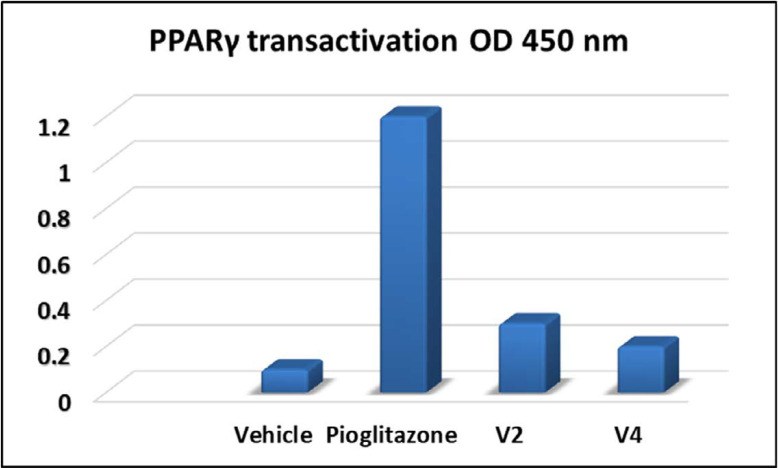
PPARγ transactivation of test compounds against pioglitazone

To reveal the PPARγ binding capability (full or partial) of the test compounds with hypoglycaemic and hypolipidemic potential, PPARγ transactivation assay was carried out by using positive control pioglitazone and DMSO was used as vehicle control. Results demonstrated that the fold activation of PPARγ by compounds V2 and V4 was 17% and 25%, respectively, compared to pioglitazone, which indicates that these compounds could be a partial PPARγ agonist (Figure 5). Thus, our results indicate that these compounds may have exhibited hypoglycaemic and hypolipidemic effects through partial activation of PPARγ.

**Scheme 1:**
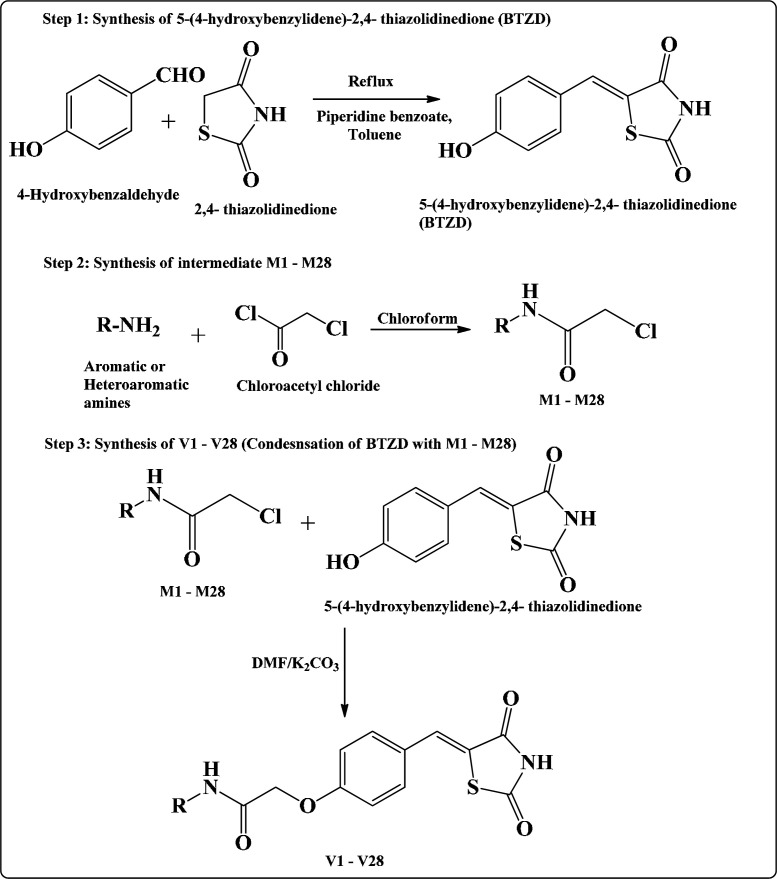
Route for the synthesis of TZD derivatives V1-V28

## Conclusion

5-benzylidene-2,4-TZDs derivatives were designed as partial PPARγ agonists with antidiabetic properties, synthesized (V1-V28), purified, and structurally characterized by using different spectral techniques such as FTIR, NMR (^1^H & ^13^C), and mass spectrometry. All these chemical compounds were found safer on normal hepatocytes, as demonstrated by *in vitro *MTT assay. Twenty compounds were further tested to determine their *in vivo* hypoglycaemic and hypolipidemic efficacy in HFD and a low dose of STZ-induced diabetic rats. Out of these twenty screened derivatives, four compounds (V2, V4, V5, and V20) have shown excellent hypoglycaemic and hypolipidemic activity (more than 80%), which is comparable to pioglitazone. Histopathology of liver and heart displayed no significant toxicity. In addition, to confirm the PPARγ agonistic activity, transactivation assay was performed for the two representative compounds V2 and V4, which revealed fold activation of 17% and 25% respectively of PPARγ as compared to pioglitazone. Thus, based on the results, we assume that these compounds could be further developed as hypoglycemic and hypolipidemic agents in the future.
